# Supplementary material for: Tead1a Initiates Transcriptional Priming Through the TEAD1a/YAP‐Notch1‐Spi1/Cebpα Axis to Promote Neutrophil Fate
Source: Adv Sci (Weinh). 2025 Aug 28;12(41):e05441. doi: 10.1002/advs.202505441 (PMC12591127; doi:10.1002/advs.202505441)
Supplement: Supplementary file 4 — Supplementary Dataset [file ADVS-12-e05441-s004.zip › 3-3.zip!/3-3/02-3.cluster/cluster6/Enrich/ego_CC.pdf]

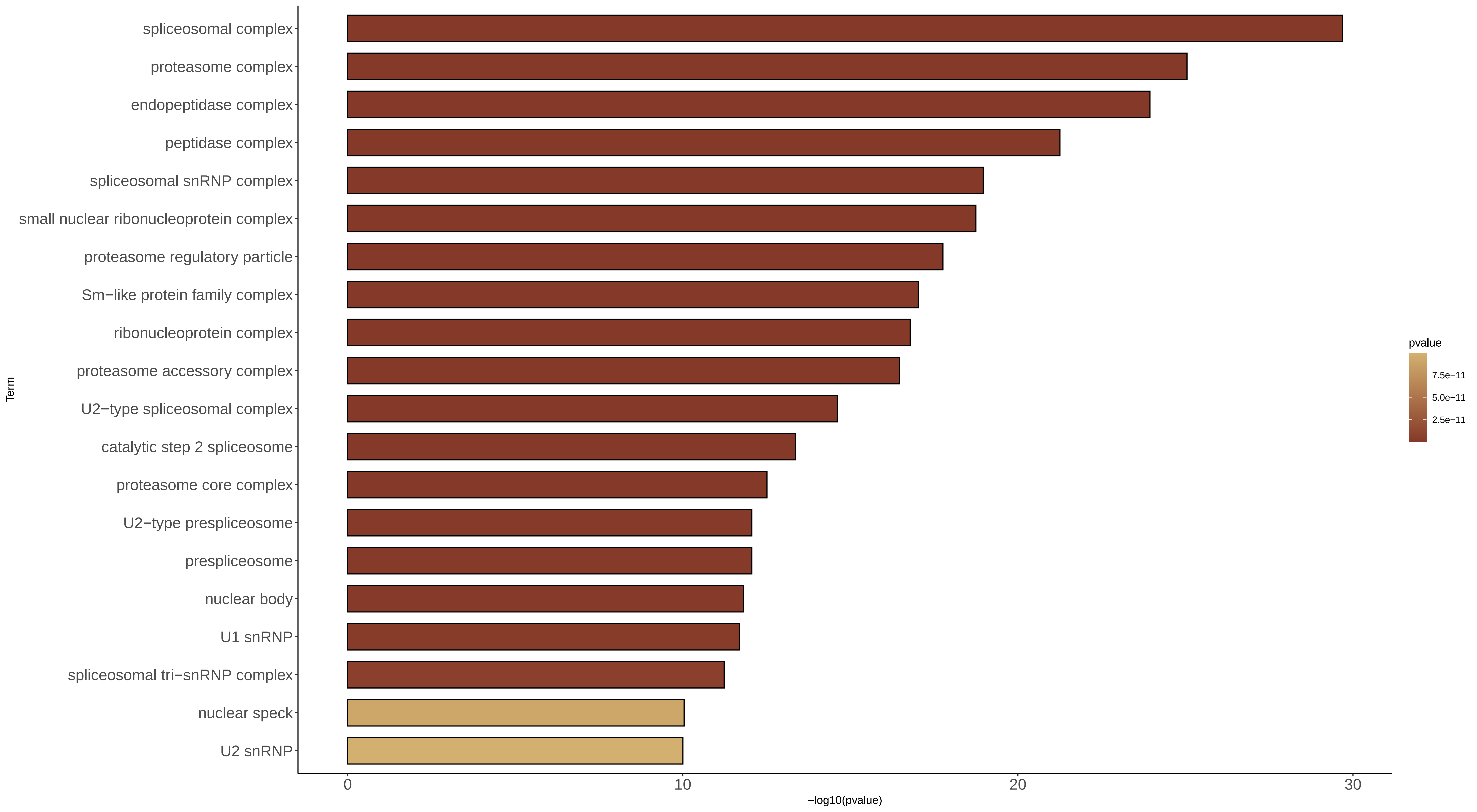
spliceosomal complex
proteasome complex
endopeptidase complex
peptidase complex
spliceosomal snRNP complex
small nuclear ribonucleoprotein complex
proteasome regulatory particle
Sm−like protein family complex
ribonucleoprotein complex
proteasome accessory complex
U2−type spliceosomal complex
catalytic step 2 spliceosome
proteasome core complex
U2−type prespliceosome
prespliceosome
nuclear body
U1 snRNP
spliceosomal tri−snRNP complex
nuclear speck
U2 snRNP
Term
0
10
20
30
−log10(pvalue)
pvalue
7.5e−11
5.0e−11
2.5e−11
